# Gastric pneumatosis in a patient with anorexia nervosa

**DOI:** 10.1002/ccr3.4155

**Published:** 2021-05-05

**Authors:** Hiroaki Nishioka

**Affiliations:** ^1^ Department of General Internal Medicine Kobe City Medical Center General Hospital Kobe Japan

**Keywords:** anorexia nervosa, gastric pneumatosis, portal vein gas

## Abstract

Gastric pneumatosis can be caused by mechanical force, pulmonary disease, bacterial infection, and ischemic condition. It can be managed conservatively, however, according to the etiology, surgery may be required.

## Graphical abstract

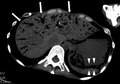

Gastric pneumatosis, air in the stomach wall, is uncommon and an ominous radiological sign, which indicates a serious gastrointestinal problem in the majority of patients. However, I herein show a patient with anorexia nervosa who developed gastric pneumatosis from intractable vomiting and recovered with conservative management.

A 42‐year‐old woman with an 18‐year history of anorexia nervosa presented with abdominal pain and repeated vomiting that lasted for 1 day. Her body mass index was 10.6. She was experiencing tenderness in the epigastric region. Blood test results were unremarkable. Abdominal computed tomography showed gas within the gastric wall and diffuse hepatic portal venous gas (Figure 1A,B). Upper endoscopy revealed exfoliation of the gastric mucosa and submucosa. A nasogastric tube was placed for gastric lavage and decompression, and lansoprazole was administered. Two days later, her abdominal pain subsided and nausea was resolved; she consumed a small meal without symptom recurrence. Abdominal computed tomography on day 9 showed a normal gastric and liver.

**FIGURE 1:**
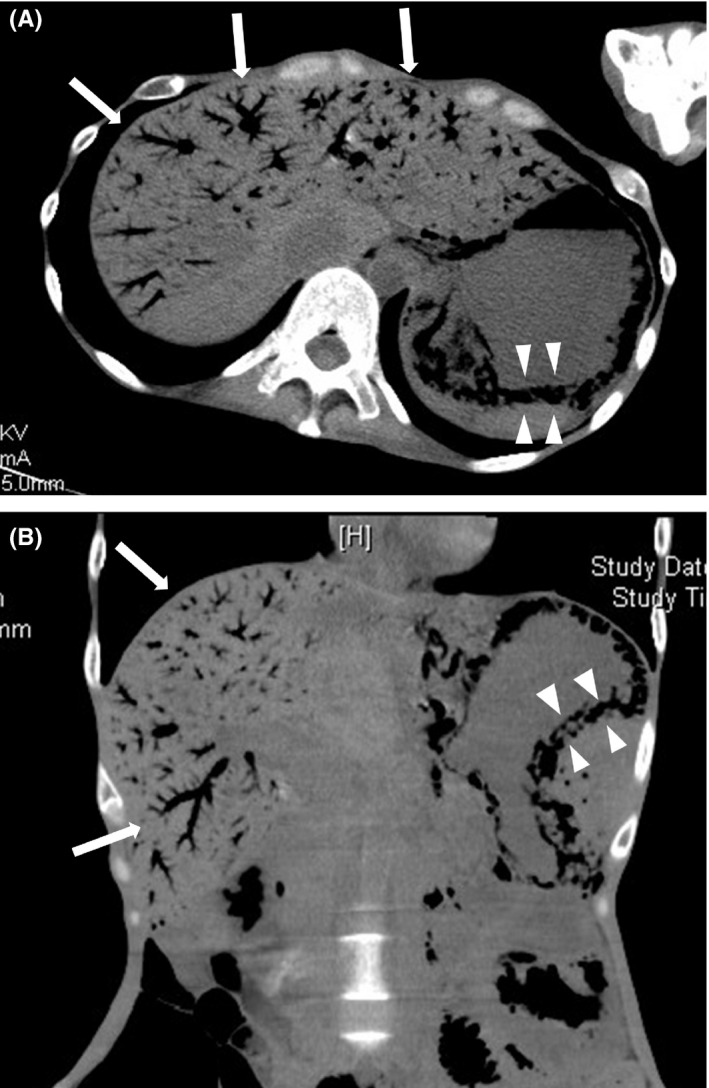
Abdominal computed tomography demonstrates gas within the gastric wall (white arrow heads) and the intrahepatic portal veins (white arrows). A, Axial view, B, Coronal view

Gastric pneumatosis can be caused by mechanical force, pulmonary disease, bacterial infection, and ischemic condition, which often act concurrently.^1^, ^2^ In this case, gastric pneumatosis may have been caused by damage to the gastric mucosa and a sudden increase in intragastric pressure due to repeated episodes of vomiting. The gastric mucosa and wall may be friable because of malnutrition caused by anorexia nervosa. Gastric pneumatosis can be managed conservatively, such as our case, however, surgery may be required, especially in gastric gangrene from infection.

## DATA AVALABILITY STATEMENT

Data sharing is not applicable to this article as no datasets were generated or analyzed during the current study.

## CONFLICT OF INTEREST

None declared.

## AUTHOR CONTRIBUTION

HN wrote the article and made the literature review.

## CONSENT STATEMENT

Published with written consent of the patient.

## ETHICAL APPROVAL

The patient gave me her agreement to publish her clinical history. This case is anonymous.
